# hUMSCs Restore Uterine Function by Inhibiting Endometrial Fibrosis via Regulation of the MMP-9/TIMP-1 Ratio in CDDP-Induced Injury Rats

**DOI:** 10.1155/2023/8014052

**Published:** 2023-03-20

**Authors:** Yu Tang, Yaru Si, Chengen Liu, Cui Li, Li Qu, Ying Liu, Qiang Fu, Qianqian Luo

**Affiliations:** ^1^College of Basic Medicine, Binzhou Medical University, Yantai, Shandong 264003, China; ^2^School of Pharmacology, Institute of Aging Medicine, Binzhou Medical University, Yantai, Shandong 264003, China; ^3^Clinical Medical School, Binzhou Medical University, Yantai, Shandong 264003, China; ^4^Yantai Affiliated Hospital of Binzhou Medical University, Yantai, Shandong 264100, China; ^5^Shandong Cellogene Medicine Science & Technology Co., Ltd., Yantai, Shandong 264003, China

## Abstract

The fertility of females of childbearing age who are cured of cancer by chemotherapy is gradually declining globally. As a broad-spectrum chemotherapy drug in clinic, the damage of cisplatin (CDDP) to female reproductive function cannot be ignored. At present, the study of CDDP damage to the uterus is not sufficient, and the exact mechanism needs to be further explored. Therefore, we conducted this research to determine whether uterine injury in CDDP-induced injury rats might be improved by human umbilical cord mesenchymal stem cells (hUMSCs) and to further explore the precise mechanism. The rat model of CDDP-induced injury was established by intraperitoneal injection of CDDP, and hUMSCs were injected into the tail vein 7 days later. *In vivo*, uterine function in CDDP-induced injury rats was affected after hUMSC transplantation. *In vitro*, the specific mechanism was further explored from the cell and protein levels. Overall, the specific reason of CDDP-induced uterine dysfunction in rats was endometrial fibrosis, which was significantly improved after hUMSC transplantation. Further investigation of the mechanism found that hUMSCs could regulate the ratio of matrix metalloproteinase-9 (MMP-9)/tissue inhibitor of metalloproteinase-1 (TIMP-1) in endometrial stromal cells (EnSCs) after CDDP injury.

## 1. Introduction

Infertility has become a global health problem and has been widely concerned by the society. This problem not only brings great economic and psychological pressure to couples of childbearing age but also has a negative impact on social harmony. Female factors account for 12%-15% of infertility problems, among which uterine dysfunction and multiple ovarian dysfunction are more common [1, 2]. According to statistics [3, 4], there are more than 1 million new cancer patients in females of childbearing age in the world every year, and 80% of them suffer from fertility decline after receiving chemotherapy. Chemotherapy is an important factor in the reduction of female fertility, and its main side effect is uterine function damage [5, 6]. Cisplatin (CDDP) is known to be one of the widely used chemotherapy drugs in clinical practice, which belongs to metal-platinum complex and cycle nonspecific antitumor drug. As a broad-spectrum anticancer drug, it is often used in the treatment of various clinical solid tumors, such as ovarian cancer, breast cancer, and lung cancer [7–9]. The pathogenesis of cisplatin has always been a hot topic in the field of female reproduction. However, the understanding of the effect of cisplatin on the uterus is still insufficient and needs to be further explored.

In recent years, mesenchymal stromal cells (MSCs) have been widely used in the treatment of various tissues and organs injuries and the potential for MSCs to repair or regenerate damaged endometrium became an important field to be investigated [10–12]. Human umbilical cord mesenchymal stem cells (hUMSCs) represent the preferred stem cells for such treatments due to their capacity for multilineage differentiation, low immunogenicity, accessibility, and the benefit on survival time after transplantation [13–15]. However, the specific mechanisms by which hUMSCs can reverse the CDDP-induced injury to uterine function remain unknown.

For uterus, decreased fertility and adverse pregnancy outcomes are mainly caused by endometrial damage, as the endometrium is the site for embryo implantation and utero placenta exchange [16]. In the endometrium, endometrial stromal cells (EnSCs) are one of the main cell types. Under normal conditions, EnSC subsets can differentiate into epithelial cells and maintain homeostasis. However, while endometrial damage happened, EnSCs can differentiate into myofibroblasts, which result in an accumulation of a large amount of extracellular matrix (ECM) [17]. It is the excessive deposition and reorganization of ECM that play an important role during endometrial fibrosis [18]. Endometrial fibrosis impairs female reproductive ability, leading to amenorrhea, abortion, infertility, and other symptoms [19].

Matrix metalloproteinase-9 (MMP-9) is a zinc-dependent endopeptidase that can selectively degrade ECM and participate in tissue remodeling. On the other hand, MMP-9 activity is regulated by tissue inhibitor of metalloproteinase-1 (TIMP-1), which is an endogenous inhibitor. The balance between matrix metalloproteinases (MMPs) and tissue inhibitor of metalloproteinases (TIMPs) represents a key factor for the normal ECM reconstruction and tissue repair [20–22], while the disruption of this balance can result in endometrial fibrosis due to excessive deposition and remodeling of the ECM [18, 23].

Therefore, we hypothesized that hUMSC transplantation may regulate the MMP-9/TIMP-1 ratio of EnSCs to reverse the CDDP-induced endometrial damage and subsequently restore uterine function.

## 2. Materials and Methods

### 2.1. Animals

Female Wistar rats at 7 weeks of age were purchased from the Shandong Pengyue Experimental Animal Breeding Co., Ltd. for use *in vivo* experiments. Rats were maintained in animal facilities with controlled temperature and humidity and had free access to water and food. All procedures of this experiment were approved by the Animal Protection and Use Committee of Binzhou Medical University. The study was performed under the National Research Council's guidance for the care and use of laboratory animals.

### 2.2. Chemicals

Cisplatin (CDDP; Meilunbio, China) in a stock solution of 3.33 mM was dissolved in distilled water (DW) and added to endometrial stromal cell cultures at final concentrations of 0-60 *μ*M.

### 2.3. Animal Models

The rats were divided into four groups and received 7 consecutive daily injections: control, CDDP, CDDP+PBS, and hUMSCs (*n* = 32/group). Rats in the control group received intraperitoneal injections of normal saline. For the CDDP group, rats received injections of CDDP (1 mg/kg, dissolved in normal saline). In the CDDP+PBS group, 300 *μ*l of phosphate-buffered saline (PBS) was injected into the tail vein after 7 days of CDDP injections. For the hUMSC group, 300 *μ*l of PBS containing 2 × 10^6^ hUMSCs was injected into the rat tail vein after 7 days of CDDP injections. In the process of hUMSC injection, it was necessary to avoid excessive injection speed leading to the death of congestive heart failure. The survival rate of rats in the hUMSC group was 100%. One week after the hUMSC injection, the rats from each group (*n* = 14) were randomly selected for fertility tests, while the remaining rats were used in subsequent studies.

### 2.4. Isolation and Culture of hUMSCs

Umbilical cords used in this experiment were obtained from healthy, full-term pregnant women who had signed informed consents. The collected umbilical cords were washed repeatedly with PBS, and both ends were removed with ophthalmic scissors and cut into 1 cm per segment. Then, the umbilical artery, umbilical vein, and umbilical capsule were dissected with ophthalmic scissors and tweezers. Ophthalmic scissors were then used to cut up the remaining tissue. The fragments were inoculated in a low-glucose medium containing 10% fetal bovine serum (FBS; Gibco, South America) and 1% penicillin-streptomycin liquid (PS; Solarbio, China). The tissue fragments were cultured at 37°C in an incubator with 5% CO_2_. After this initial culture, the solution was changed every 3.5 days and, when the tissue mass appeared as a clone ball, the entire solution was changed. When cells had grown to 80-90%, they were passaged. Cell passages of 3-5 generations were used for later experiments. To identify hUMSCs, Alizarin Red and Oil Red O staining were used to test for their ability to differentiate into osteoblasts and adipocytes, respectively. Cell surface markers consisting of CD44, CD73, CD90, CD34, HLA-DR, and CD45 were determined using flow cytometry.

### 2.5. Characterization of hUMSCs

The hUMSCs from the third to fifth generations were used for phenotypic identification. They were grown in 6-well plates and digested and centrifuged when the cell density reached about 80%. The centrifuged cells were resuspended with PBS, divided into appropriate portions, and added to the corresponding flow tubes, with about 100 *μ*l of cell suspension per flow tube. Except blank tubes, the other flow tubes were filled with the corresponding flow antibodies of FITC-labeled mice anti-human CD73mAb, CD90mAb, and CD44mAb and PE-labeled mice anti-human HLA-DRmAb, CD34mAb, and CD45mAb. The cells were incubated at 4°C for 30 min without light and centrifuged. Then, the supernatant was removed, and 300 *μ*l PBS was added to resuspend the cells for flow cytometry analysis.

### 2.6. Differentiation of hUMSCs

The hUMSCs from the first three generations were used for differentiated identification. They were cultured in 24-well plates, and the original medium was removed when the cell density reached about 90%. Osteogenic and adipogenic differentiation kits (STEMCELL Technologies, Canada) were used for hUMSC differentiation induction. The cells were cultured for 28 days and 14 days and stained with Alizarin Red and Oil Red O, respectively. Then, the cells were fixed with 4% paraformaldehyde for 30 min and washed with PBS. The differentiation of hUMSCs could be observed under an optical microscope.

### 2.7. Culture of hEnSCs

The human endometrial stromal cells (hEnSCs) purchased from Wuhan Churuike Pharmaceutical Technology Co., Ltd. (CRK Pharma, China) were incubated in DMEM-F12 medium (HyClone, USA) containing 10% fetal bovine serum (FBS, AusGeneX, Australia) and 1% penicillin and streptomycin (PS; Solarbio, China). Cells were then cultured in a 5% CO_2_ incubator with saturated humidity at 37°C. Cell growth was monitored daily, and cell passages were conducted every 3 days.

### 2.8. Hematoxylin and Eosin (H&E) Staining

Samples of uterine tissue from each group were obtained and fixed in 4% paraformaldehyde for 24 h. They were then embedded in paraffin, sliced and baked, dewaxed to water, and stained with H&E. Morphology, number of glands, and endometrial thickness of uterus were measured under light microscopy.

### 2.9. Masson Trichrome Staining

Uterine samples were soaked in 4% paraformaldehyde for 24 h. The samples were then dehydrated, embedded, sliced, baked, and subjected to Masson Trichrome staining (Solarbio, China). Stained tissue samples were photographed and observed under light microscopy to evaluate the degree of endometrial fibrosis in each group.

### 2.10. Immunofluorescent Staining

Expressions of Collagen I, Collagen III, CTGF, Fibronectin, *α*-SMA, MMP-9, and TIMP-1 proteins were determined with using immunofluorescent staining. To observe hEnSCs or uterine fibrosis and the ratio between MMP-9 and TIMP-1, the slides were washed 3 times with PBS, after which they were embedded in PBS containing 5% donkey serum for 30 min (Santa Cruz Biotechnology, USA). The hEnSCs and uterine sections were incubated with primary antibodies consisting of anti-Collagen I (1 : 100; Proteintech, China), anti-Collagen III (1 : 100; Proteintech, China), anti-CTGF (1 : 100; Proteintech, China), anti-Fibronectin (1 : 200; ABclonal, China), anti-*α*-SMA (1 : 100; Abcam, UK), anti-MMP-9 (1 : 200; Affinity, China), or anti-TIMP-1 (1 : 200, Affinity, China) at 4°C overnight. On the following day, the hEnSCs or uterine sections were maintained at room temperature for 1 hour and then incubated with secondary antibodies consisting of goat anti-rabbit IgG, Alexa Fluor 549 (Invitrogen, USA), or a mixture of goat anti-rabbit IgG and Alexa Fluor 549 and goat anti-mouse IgG and Alexa Fluor 488 (Invitrogen, USA). The hEnSCs or uterine sections were then incubated with DAPI (Solarbio, China) at room temperature and away from light for 10 min. The staining of hEnSCs or uterine sections was visualized using an inverted fluorescent microscope (Leica, Germany).

### 2.11. Immunohistochemistry

Uterine estrogen and progesterone receptors and endometrial receptivity were assessed by staining paraffin sections with anti-estrogen and anti-progesterone receptors and anti-HOXA10 antibody. Uterine sections were incubated with the primary antibodies for the anti-estrogen receptor (1 : 200; Bioss, China), anti-progesterone receptor (1 : 200; Bioss, China), or anti-HOXA10 (1 : 200; Bioss, China) at 4°C overnight. The slides were developed with diaminobenzidine (DAB) as chromogen and then counterstained with hematoxylin. Stained slides were viewed under a microscope, and the German immunoreactive score (IRS) was used to analyze staining results.

### 2.12. Enzyme-Linked Immunosorbent Assay (ELISA)

Uterine tissue homogenates were extracted from each group, and levels of Collagen I, Collagen III, CTGF, Fibronectin, and *α*-SMA expression were detected with the use of ELISA kits (Mlbio, China).

### 2.13. Scanning Electron Microscopy (SEM)

Uterus from each group was fixed with 2.5% glutaraldehyde for 24 h. After dehydration, drying, and gold spraying, the pinopodes were observed using scanning electron microscopy (SEM).

### 2.14. Fertility Examination

Fourteen rats were randomly selected from each group and were mated with sexually mature males in a ratio of 2 : 1. Inspections for vaginal plugs were conducted at 08:00 am each day to determine mating success. After the appearance of a vaginal plug, female rats were separated from the males and birth of rat pups was observed approximately 20 days later. The number of rat pups and pregnancy rates of each group were recorded.

### 2.15. Western Blot

Uterine tissue and cultured hEnSCs were lysed using radioimmunoprecipitation (RIPA) buffer, and protein concentrations were determined using a bicinchoninic acid assay (Solarbio, China). Cell and tissue samples were electrophoresed on sodium dodecyl sulfate polyacrylamide gel followed by transfer to PVDF membranes. After blocking with 5% skim milk, the membranes were incubated with anti-Collagen I (1 : 2000; Proteintech, China), anti-Collagen III (1 : 1500; Proteintech, China), anti-CTGF (1 : 800; Proteintech, China), anti-Fibronectin (1 : 800; ABclonal, China), anti-*α*-SMA (1 : 1000; Abcam, UK), anti-MMP-9 (1 : 1000; Affinity, China), anti-TIMP-1 (1 : 1000; Affinity, China), or anti-GAPDH (1 : 20000; Proteintech, China) polyclonal antibodies at 4°C overnight. On the following day, membranes were washed with TBS and Tween 20 (TBST) and then immunoblotted with HRP-conjugated secondary antibodies (Proteintech, China) for 1 h at room temperature. Expressions of each protein were determined using the enhanced chemiluminescence reagent (ECL) kit (Sparkjade Science Co., Ltd., China), and band densities were measured with using ImageJ software.

### 2.16. CCK-8 Cell Viability Assay

The CCK-8 kit (Meilong Bio, China) was used to evaluate the effects of different concentrations of CDDP on hEnSC activity. Cells (5 × 10^3^ cells/well) were seeded onto 96-well plates and cultured overnight. After cell adhesion, the medium was replaced with one containing CDDP (0-60 *μ*m). Twenty-four hours later, the CCK-8 working solution (10 *μ*l) was added to the cell culture medium for 1 h and the absorbance was measured at 450 nm.

### 2.17. Data Analysis

Quantitative data are presented as the means ± SDs, and SPSS 23.0 software was used for data analysis. One-way analysis of variance (ANOVA) with the post hoc Bonferroni test was used to assess differences among the groups. Qualitative data are presented as rates, and chi-square tests were used for comparisons between groups. A *P* < 0.05 was required for results to be considered statistically significant.

## 3. Results

### 3.1. Characteristics and Differentiation of hUMSCs

As shown in Supplemental Figure (available here), the morphology of isolated cells resembled fibroblasts and could be differentiated to osteoblasts and adipocytes, showing positive staining for Alizarin Red S and Oil Red O, respectively. The FCM results showed that the isolated cells expressed CD44, CD73 and CD90, but not CD45, CD34, or HLA-DR. These results are consistent with those reported in our previous studies [24, 25].

### 3.2. hUMSC Transplantation Improved Uterine Morphology in CDDP-Induced Injury Rats

**Figure 1:**
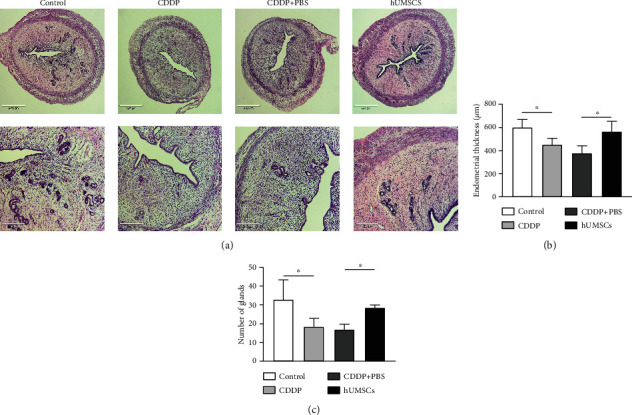
Uterine morphological characteristics. (a) Uterine tissue was examined microscopically following H&E staining in each group (40×, 100×). (b) Changes in endometrial thickness in each group. (c) Number of endometrial glands in the uterus of each group. Data are presented as the means ± SDs. ^∗^*P* < 0.05,  ^∗∗^*P* < 0.01, and^∗∗∗^*P* < 0.001. CDDP: cisplatin; H&E: hematoxylin and eosin.

H&E staining was used to assess the effect of hUMSC transplantation on uterine morphology in CDDP-induced injury rats. As shown in Figure 1(a), the endometrial thickness gets significantly thinner in the CDDP group (*P* < 0.05; Figure 1(b)). After hUMSC transplantation, endometrial thickness was restored and thicker than that in the CDDP+PBS group (*P* < 0.05; Figure 1(b)). The number of endometrial glands was reduced in the CDDP group as compared with the control group (*P* < 0.05; Figure 1(c)). However, at 7 days after hUMSC injection, the number of endometrial glands was increased over that in the CDDP+PBS group (*P* < 0.05; Figure 1(c)). These results showed that hUMSC transplantation restored CDDP-induced uterine morphological injury.

### 3.3. hUMSC Transplantation Restored Uterine Function in CDDP-Induced Injury Rats

**Figure 2:**
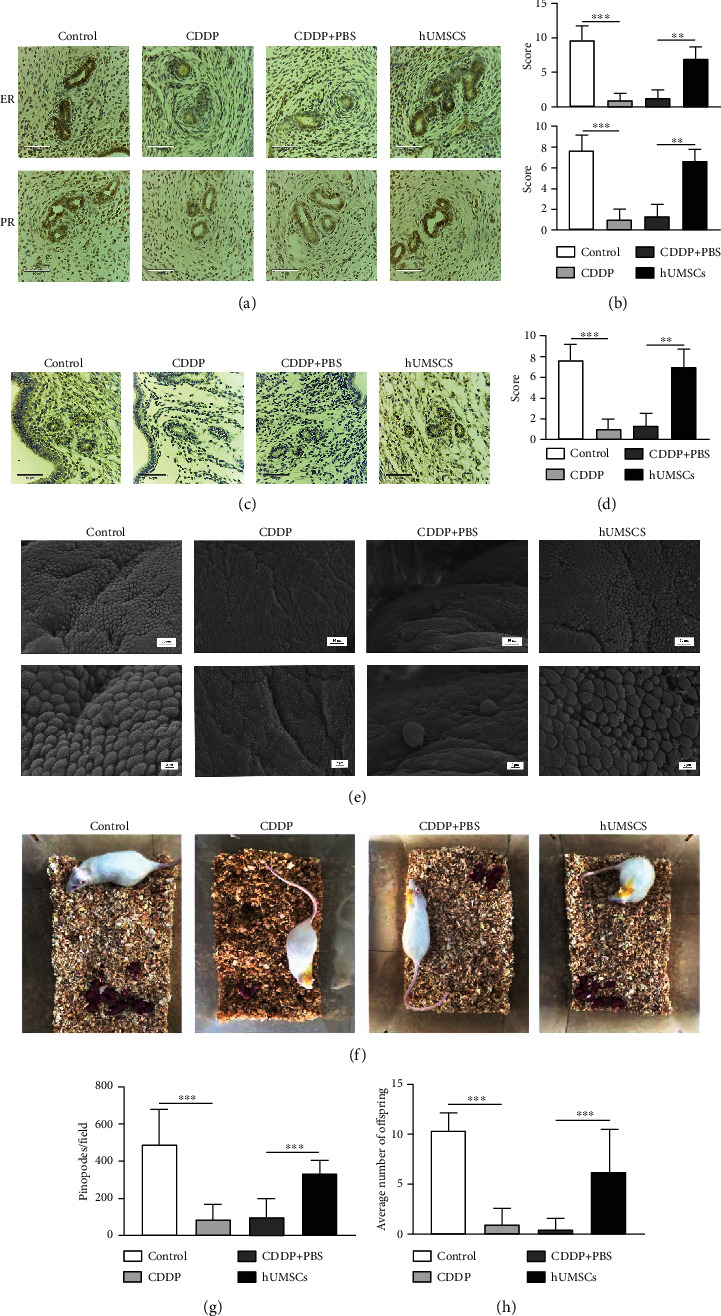
Changes of ER, PR, and receptivity in endometrium. (a) Immunohistochemical staining was used to observe the expressions of ER and PR in uterine tissues of each group (200×). (b) Intensities of ER and PR staining within each group as quantified using ImageJ. (c) Representative examples of HOXA10 staining within uterine tissue in each group (200x). (d) Intensities of HOXA10 staining within each group as quantified were using ImageJ. (e) Pinopodes as observed with use of scanning electron microscopy (1.00KX, 3.00KX). (f) Fertility rates of rats in each group. (g) Summary of total numbers of endometrial pinopodes per field. (h) Summary of the average number of litter sizes in each group. Data are presented as the means ± SDs. ^∗^*P* < 0.05,  ^∗∗^*P* < 0.01, and^∗∗∗^*P* < 0.001. CDDP: cisplatin; ER: estrogen receptor; PR: progesterone receptor; HOXA10: homolobox A10.

In order to investigate the effect of hUMSC transplantation on uterine function in CDDP-induced injury rats, a number of assays were performed including expression of estrogen and progesterone receptors and HOXA10 with immunohistochemical staining, pinopodes as observed using scanning electron microscopy; we also recorded the litter size number and pregnancy rates in each group. As shown in Figures 2(a) and 2(b), levels of estrogen and progesterone receptors within the uterus of the CDDP group were markedly decreased as compared with that of the control group (*P* < 0.001), while hUMSC transplantation in CDDP-induced injury rats restored the levels of these receptors (*P* < 0.01). Results from uterine immunohistochemical staining showed that the expression of HOXA10 was decreased in the CDDP group compared with the control group (*P* < 0.001), whereas, in response to hUMSC transplantation, HOXA10 expression was increased (*P* < 0.01; Figures 2(c) and 2(d)). The changes of morphology and number in endometrial pinopodes were observed by scanning electron microscopy. In the control group, the endometrial surface was regular and the pinopodes were abundant and similar in shape and size, whereas small and varied shapes and sizes of pinopodes were observed in the endometrium of CDDP-induced injury rats (*P* < 0.001). After hUMSC transplantation, the endometrial surface in CDDP-induced injury rats became more regular, with significant increases in the number of pinopodes, and clearly reduced gaps were present (*P* < 0.001; Figures 2(e) and 2(g)). As shown in Figures 2(f) and 2(h), litter sizes in the CDDP group were smaller than those in the control group (*P* < 0.001), while hUMSC transplantation remarkably increased litter sizes than the CDDP+PBS group (*P* < 0.001). In addition, the results as presented in Table 1 show that pregnancy rates of the CDDP-induced injury rats were significantly increased after hUMSC transplantation (*P* < 0.01). Collectively, these results suggest that hUMSC transplantation can restore uterine function in CDDP-induced injury rats.

### 3.4. hUMSC Transplantation Relieved Uterine Fibrosis in CDDP-Induced Injury Rats

**Figure 3:**
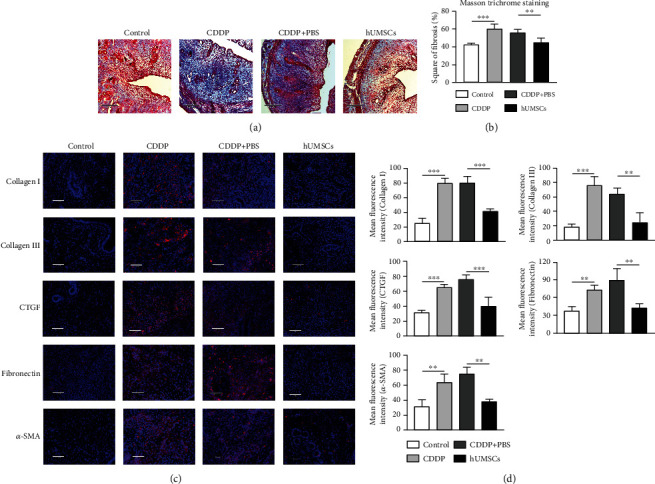
Histopathological analysis of uterine fibrosis. (a) Masson trichrome staining of uterine tissue in each group as observed under microscopy (100x). (b) Masson trichrome staining scores were quantitated using ImageJ software. (c) Immunofluorescent staining of Collagen I, Collagen III, CTGF, Fibronectin, and *α*-SMA expressions in uterine tissues of each group (200x). (d) Mean fluorescent intensities as analyzed by ImageJ software. Data are presented as the means ± SDs. ^∗^*P* < 0.05,  ^∗∗^*P* < 0.01, and^∗∗∗^*P* < 0.001. CDDP: cisplatin; Collagen I: Collagen Type I; Collagen III: Collagen Type III; CTGF: connective tissue growth factor; *α*-SMA: alpha smooth muscle actin.

**Figure 4:**
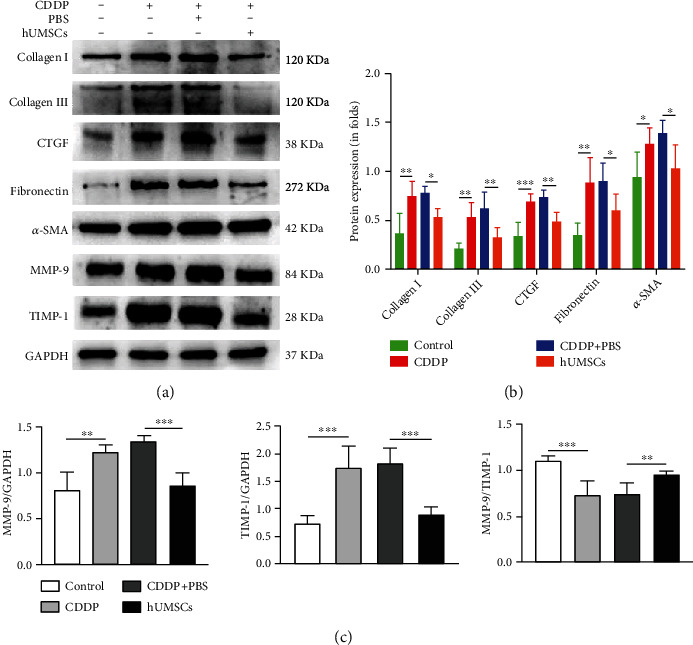
Western blot analyses of uterine tissue fibrosis-related, MMP-9, and TIMP-1 proteins. (a) Uterine expressions of Collagen I, Collagen III, CTGF, Fibronectin, *α*-SMA, MMP-9, and TIMP-1 in each group. A representative blot from one of the three independent experiments performed is shown. GAPDH was used as the loading control. (b) Expressions of Collagen I, Collagen III, CTGF, Fibronectin, and *α*-SMA protein as quantitated using ImageJ software. (c) Levels of MMP-9, TIMP-1, and the MMP-9/TIMP-1 ratio in each group. Data are presented as the means ± SDs. ^∗^*P* < 0.05,  ^∗∗^*P* < 0.01, and^∗∗∗^*P* < 0.001. CDDP: cisplatin; Collagen I: Collagen Type I; Collagen III: Collagen Type III; CTGF: connective tissue growth factor; *α*-SMA: alpha smooth muscle actin; MMP-9: matrix metalloprotein 9; TIMP-1: tissue inhibitor of metalloproteinase-1; GAPDH: glyceraldehyde-3-phosphate dehydrogenase.

**Figure 5:**
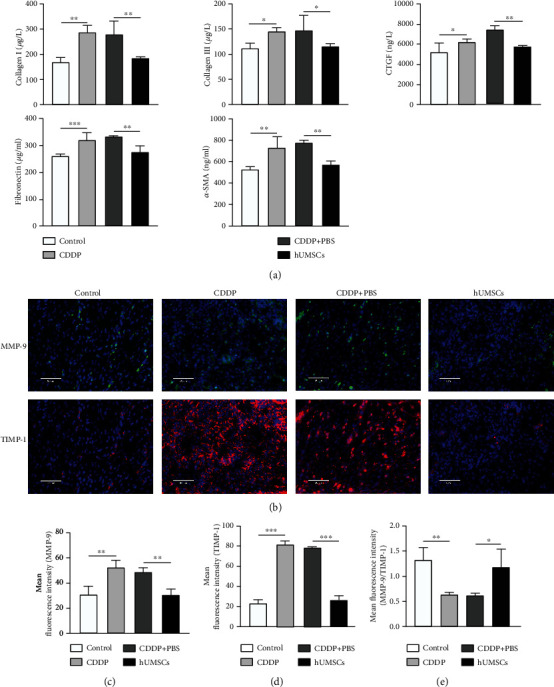
ELISA and immunofluorescent staining of uterine expressions of fibrosis-related, MMP-9, and TIMP-1 proteins. (a) Levels of Collagen I, Collagen III, CTGF, Fibronectin and *α*-SMA in each group. (b) Immunofluorescent staining of MMP-9 and TIMP-1 expressions in uterine tissues of each group (200x). (c–e) Mean fluorescent intensities as analyzed by ImageJ software. Data are presented as the means ± SDs. ^∗^*P* < 0.05,  ^∗∗^*P* < 0.01, and^∗∗∗^*P* < 0.001. CDDP: cisplatin; Collagen I: Collagen Type I; Collagen III: Collagen Type III; CTGF: connective tissue growth factor; *α*-SMA: alpha smooth muscle actin; MMP-9: matrix metalloprotein 9; TIMP-1: tissue inhibitor of metalloproteinase-1.

As the Masson trichromatic staining results show in Figures 3(a) and 3(b), the area of uterine fibrotic tissue was increased in the CDDP group (*P* < 0.001) compared with the control group. After hUMSC transplantation, fibrotic areas of uterine tissue were observably reduced (*P* < 0.01). Results from immunofluorescent staining showed that Collagen I, Collagen III, CTGF, Fibronectin, and *α*-SMA fibrosis protein expression all dramatically increased in the CDDP group versus the control group (*P* < 0.01). After hUMSC transplantation, fluorescent intensities of Collagen I, Collagen III, CTGF, Fibronectin, and *α*-SMA in the CDDP group were significantly reduced (*P* < 0.01; Figures 3(c) and 3(d)). In addition, results from western blot and ELISA showed that, compared with the control group, Collagen I, Collagen III, CTGF, Fibronectin, and *α*-SMA expression in the CDDP group dramatically increased (*P* < 0.05). The fibrotic protein in the uterus of CDDP-induced injury rats was markedly reduced after hUMSC transplantation (*P* < 0.05; Figures 4(a), 4(b), and 5(a)). These results are consistent with the immunofluorescent staining. Furthermore, western blot and immunofluorescent staining were performed to measure the MMP-9 and TIMP-1 expression. These assays showed that compared with the control group, protein expression and fluorescent intensity of MMP-9 and TIMP-1 were increased in the CDDP group (*P* < 0.01), and the ratio of MMP-9/TIMP-1 was decreased (*P* < 0.01). After hUMSC transplantation, these protein expressions and fluorescent intensities of MMP-9 and TIMP-1 were significantly decreased (*P* < 0.01), and the MMP-9/TIMP-1 ratio was increased (*P* < 0.05; Figures 4(a), 4(c), and 5(b)–5(e)). Therefore, hUMSC transplantation effectively inhibited the induction of fibrosis within the uterus of CDDP-induced injury rats. This effect may be related to the balance between MMP-9 and TIMP-1, with the changes in MMP-9/TIMP-1 ratio resulting after hUMSC transplantation contributing to the recovery of uterine function in CDDP-induced injury rats.

### 3.5. hUMSCs Regulated the Ratio of MMP-9/TIMP-1 to Inhibit Fibrosis in hEnSCs

**Figure 6:**
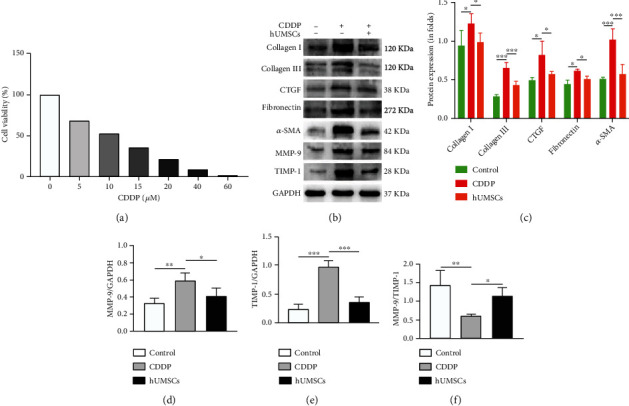
Fibrosis, MMP-9, and TIMP-1 expressions in hEnSCs. (a) Cell viability of hEnSCs as determined in response to varying concentrations of CDDP. (b) Expressions of Collagen I, Collagen III, CTGF, Fibronectin, *α*-SMA, MMP-9, and TIMP-1 in hEnSCs within each group. A representative blot from one of the three independent experiments performed is shown. GAPDH was used as the loading control. (c) Levels of Collagen I, Collagen III, CTGF, Fibronectin, and *α*-SMA within each group. (d–f) Levels of MMP-9, TIMP-1, and MMP-9/TIMP-1 ratio within each group. Data are presented as the means ± SDs. ^∗^*P* < 0.05,  ^∗∗^*P* < 0.01, and^∗∗∗^*P* < 0.001. CDDP: cisplatin; Collagen I: Collagen Type I; Collagen III: Collagen Type III; CTGF: connective tissue growth factor; *α*-SMA: alpha smooth muscle actin; MMP-9: matrix metalloprotein 9; TIMP-1: tissue inhibitor of metalloproteinase-1; GAPDH: glyceraldehyde-3-phosphate dehydrogenase.

**Figure 7:**
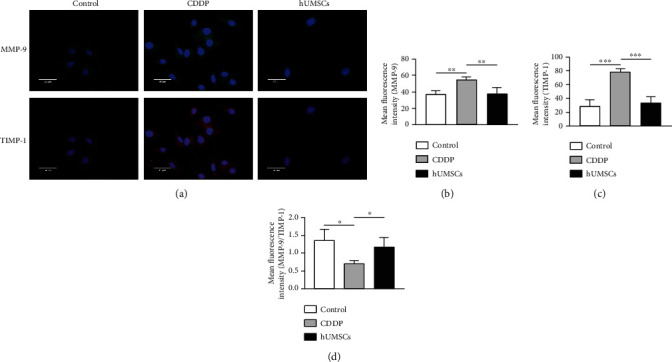
Immunofluorescent staining of MMP-9 and TIMP-1 in hEnSCs within each group. (a) MMP-9 and TIMP-1 expressions in hEnSCs within each group were determined using immunofluorescent staining (200x). (b–d) Mean fluorescent intensities were analyzed using ImageJ software. Data are presented as the means ± SDs. ^∗^*P* < 0.05,  ^∗∗^*P* < 0.01, and^∗∗∗^*P* < 0.001. CDDP: cisplatin; MMP-9: matrix metalloprotein 9; TIMP-1: tissue inhibitor of metalloproteinase-1.

We then investigated whether hUMSC transplantation could affect hEnSCs, in which way can prevent fibrosis by regulating MMP-9 and TIMP-1. To find out an appropriate concentration of CDDP on hEnSCs, a CCK-8 kit was used to detect cell viability in response to different CDDP concentrations. The 10 *μ*M CDDP was finally selected which has a cell survival rate of 54.28 ± 4.53% (Figure 6(a)). Western blot showed that the expression of Collagen I, Collagen III, CTGF, Fibronectin, and *α*-SMA was increased after CDDP treatment (*P* < 0.05), while the fibrotic protein expression was observably decreased after the hUMSC medium treatment (*P* < 0.05; Figures 6(b) and 6(c)). In addition, western blot and immunofluorescent staining were performed to measure MMP-9 and TIMP-1 (Figures 6(b), 6(d)–6(f), and 7). In the CDDP group, protein expression and fluorescent intensity of MMP-9 and TIMP-1 were increased (*P* < 0.01), but the MMP-9/TIMP-1 ratio was decreased (*P* < 0.05). Following the hUMSCs medium treatment, protein expression and fluorescent intensity of MMP-9 and TIMP-1 were decreased (*P* < 0.05) and MMP-9/TIMP-1 ratio increased (*P* < 0.05). Taken together, these data suggest that a disruption in the balance between MMP-9 and TIMP-1 might be involved in inducing fibrosis within hEnSCs.

## 4. Discussion

The hUMSCs can survive in different organs in rats, such as the lungs, hippocampus, and bones [26–28]. In our previous study, we found that hUMSC transplantation could improve endometrial receptivity in mice with CDDP injury, but we did not explore the specific reasons and mechanisms adequately [29]. Therefore, the purpose of this study was to determine whether hUMSCs could restore uterine function caused by the extensively using of the chemotherapy drug, CDDP. However, the role of hUMSCs in this therapeutic process has not been explored. It has been reported that MSCs exert their therapeutic effects in damaged tissue in two main ways: through cell replacement and cell empowerment [30]. Therefore, the following researches will focus on the specific therapeutic mechanism of hUMSCs.

With regard to the uterus, morphological and receptivity changes represent fundamental indicators of function [31]. According to previous reports, the HOXA10 gene has been proven to be a molecular marker for endometrial function and plays an important role in establishing endometrial receptivity [32]. The results of this study showed that hUMSC transplantation can recover the uterine morphology and function as shown by endometrial thickening and increases in gland number, effects which were accompanied with increased expression of endometrial HOXA10. Results from scanning electron microscopy also showed that increases in pinopodes were present in the endometrium following hUMSC transplantation, providing further support for an improvement in the endometrium. As it has been reported that ER and PR play important roles in pregnancy and endometrial repair [33], we also examined these factors in our study and found that both ER and PR expressions in the endometrium, as well as fertility, were increased after hUMSC transplantation. Collectively, these data provide substantial and robust evidence that demonstrate hUMSCs can promote endometrial repair and improve function in CDDP-induced injury rats.

Endometrial fibrosis is mainly caused of excessive deposition and recombination of ECM [34]. EnSCs, which represent an important cell type within the endometrium, are closely related with the development of fibrosis. This might contribute to the capacity of EnSCs to be transformed into myofibroblasts following endometrial injury or pathology, which leads to an accumulation of ECM and ultimately to organ fibrosis [35, 36]. Our results suggest that hUMSC transplantation can reduce uterine tissue fibrosis in CDDP-induced injury rats. In this study, we observed a decrease in fibrotic areas within the endometrium after hUMSC transplantation and found that expression of the fibrotic factors, such as ɑ-SMA, Collagen I, Collagen III, CTGF, and Fibronectin, was decreased after hUMSC transplantation. Similarly, in in vitro cell culture studies, hUMSC treatment managed to decrease the expression of *α*-SMA, Collagen I, Collagen III, CTGF, and Fibronectin. These results suggested that hUMSC transplantation can reduce fibrosis within the endometrium by promoting endometrial stromal cell transformation and thus restore uterine function.

MMP-9 and TIMP-1 are known as their important roles in the development of tissue fibrosis [37] and have been shown to be involved with fibrosis in multiple organs [38–40]. MMP-9 can selectively degrade ECM, resulting in tissue remodeling. In endometriosis, the expression of MMP-9 is elevated in the endometrium, enhancing the ability of EnSCs to migrate and implant [41]. This exacerbates pathological changes in the endometrium. However, its activity is inhibited by its endogenous inhibitor, TIMP-1, which prevents MMP-9 from overdegradation of the ECM. MMPs regulate ECM degradation to invade adjacent tissues in endometriosis, the MMP/TIMP ratio is increased compared with healthy endometriosis tissue [42]. Thus, MMP-9 plays a critical role in the endometrium. In our study, we found the expression of MMP-9 and TIMP-1 was significantly increased and the ratio of MMP-9 to TIMP-1 decreased in the uterus of CDDP-induced injury rats. These findings strongly support that MMP-9 and TIMP-1 are involved in uterine tissue fibrosis within CDDP-induced injury rats, which is consistent with results from previous studies indicating that MMP-9/TIMP-1 ratio is important in the pathogenesis of tissue fibrosis. Following hUMSC transplantation, the expression of MMP-9 and TIMP-1 in the uterus of CDDP group was decreased and MMP-9/TIMP-1 ratio was increased, effects of which were accompanied with significant reductions in endometrial fibrosis. All these results, in vitro and in vivo models, indicated that hUMSC transplantation caused the ratio of MMP-9/TIMP-1 in EnSCs after CDDP injury from imbalance to equilibrium, thereby inhibiting endometrial fibrosis and promoting the recovery of uterine function. As a consequence, these findings provide a novel therapeutic approach and strategy for the recovery of uterine function in surviving women of reproductive age who receive CDDP chemotherapy.

## 5. Conclusion

The results of this study demonstrate that hUMSC transplantation restores uterine function in a rat model of CDDP-induced injury. The recovery of uterine function may be related to the regulation of MMP-9/TIMP-1 in EnSCs from imbalance to balance and then inhibit endometrial fibrosis. The results of this study provide new insights into novel therapeutic targets and strategies which can be used to promote uterine function restoration and improve fertility in female chemotherapy patients of childbearing age.

## Figures and Tables

**Table 1:** Pregnancy rates of rats in each group.

Group	Pregnancy	No pregnancy	Pregnancy rate (%) (*n*/*n*)	*χ* ^2^ value	*P* value
Control	14	0	100% (14/14)	15.556^a^	0.001
CDDP	4	10	28.57% (4/14)		
CDDP+PBS	2	12	14.29% (1/14)	9.333^b^	0.002
hUMSCs	10	4	71.43% (10/14)		

hUMSCs: human umbilical cord-derived mesenchymal stem cells. Notes: pregnancy rate = total number of pregnancy/total number of matings. ^a^Control group versus CDDP group. ^b^hUMSC group versus CDDP+PBS group.

## Data Availability

All data generated and/or analyzed during this study are included in this published article.

## Abbreviations

ECM: Extracellular matrix
hUMSCs: Human umbilical cord-derived mesenchymal stem cells
MMP-9: Matrix metalloproteinase-9
TIMP-1: Tissue inhibitor of metalloproteinase-1
CDDP: Cisplatin
ELISA: Enzyme-linked immunosorbent assay analysis
H&E: Hematoxylin and eosin
ER: Estrogen receptor
PR: Progesterone receptor
HOXA10: Homolobox A10
EnSCs: Endometrial stromal cells
*α*-SMA: Alpha smooth muscle actin
Collagen I: Collagen Type I
Collagen III: Collagen Type III
CTGF: Connective tissue growth factor
MSCs: Mesenchymal stromal cells
DW: Distilled water
PBS: Phosphate-buffered saline
hEnSCs: Human endometrial stromal cells
FBS: Fetal bovine serum
PS: Penicillin-streptomycin
SDS - PAGE: Sodium dodecyl sulfate polyacrylamide gel electrophoresis
PVDF: Polyvinylidene fluoride
TBST: TBS added with Tween 20
DAB: Diaminobenzidine
SEM: Scanning electron microscopy
RIPA: Radioimmunoprecipitation
DAPI: 4,6-Diamino-2-phenyl indole
SD: Standard deviation
ANOVA: One-way analysis of variance
FCM: Flow cytometry
GAPDH: Glyceraldehyde-3-phosphate dehydrogenase
ECL: Enhanced chemiluminescence reagent.

## Supplementary Materials

Supplementary Materials Characterization and differentiation of hUMSCs. A Microscopic morphology of hUMSCs (40x). B, C hUMSCs were stained with Alizarin Red S and Oil Red O to demonstrate their differentiation into osteoblasts and adipocytes, respectively (400x, 100x). D The following surface markers of hUMSCs were identified: HLA–DR, CD34, CD45, CD73, CD90, and CD44.
