# Supplementary figures and images for: hUMSCs Restore Uterine Function by Inhibiting Endometrial Fibrosis via Regulation of the MMP-9/TIMP-1 Ratio in CDDP-Induced Injury Rats
Source: Stem Cells Int. 2023 Mar 20;2023:8014052. doi: 10.1155/2023/8014052 (PMC10042641; doi:10.1155/2023/8014052)

A

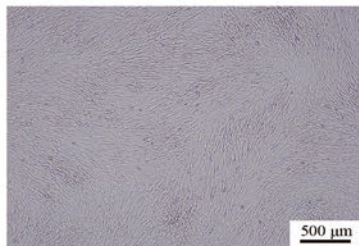

B

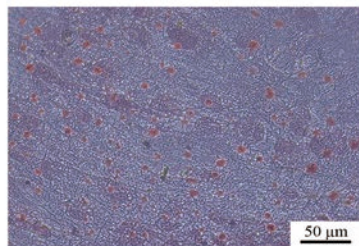

C

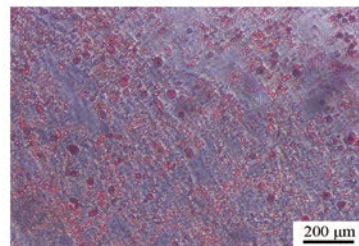

D

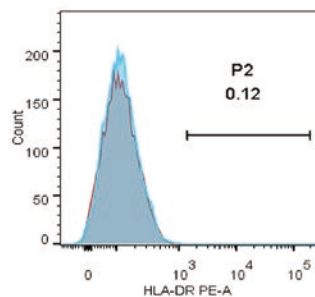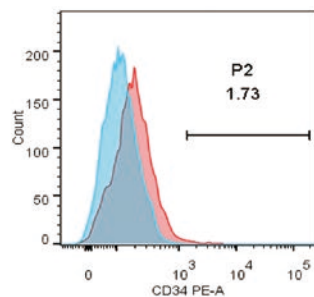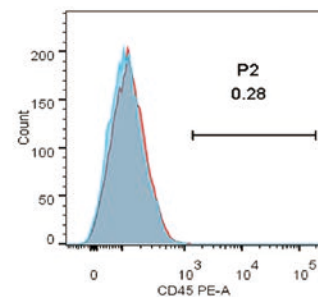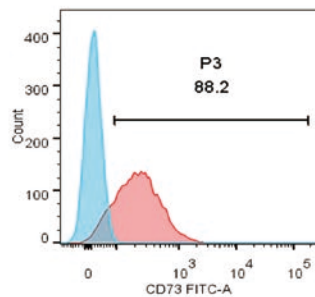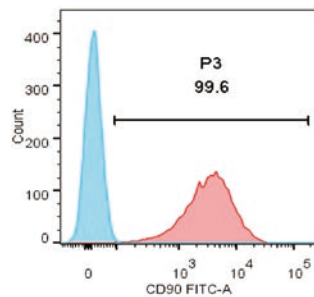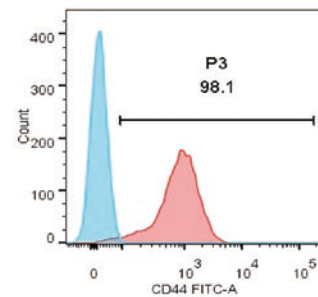

Supplement: Supplementary Materials — Characterization and differentiation of hUMSCs. A Microscopic morphology of hUMSCs (40x). B, C hUMSCs were stained with Alizarin Red S and Oil Red O to demonstrate their differentiation into osteoblasts and adipocytes, respectively (400x, 100x). D The following surface markers of hUMSCs were identified: HLA–DR, CD34, CD45, CD73, CD90, and CD44. [file 8014052.f1.pdf]
